# Links between the Brain and Retina: The Effects of Cigarette Smoking-Induced Age-Related Changes in Alzheimer’s Disease and Macular Degeneration

**DOI:** 10.3389/fneur.2016.00119

**Published:** 2016-07-27

**Authors:** Sha Sha Yu, Xin Tang, Yuen-Shan Ho, Raymond Chuen-Chung Chang, Kin Chiu

**Affiliations:** ^1^Laboratory of Retina Brain Research, Department of Ophthalmology, LKS Faculty of Medicine, The University of Hong Kong, Hong Kong, China; ^2^Tianjin Medical University, Tianjin, China; ^3^Tianjin Eye Hospital, Tianjin, China; ^4^Faculty of Health and Social Sciences, School of Nursing, The Hong Kong Polytechnic University, Hong Kong, China; ^5^Laboratory of Neurodegenerative Diseases, LKS Faculty of Medicine, School of Biomedical Sciences, The University of Hong Kong, Hong Kong, China; ^6^Research Centre of Heart, Brain, Hormone and Healthy Aging, The University of Hong Kong, Hong Kong, China; ^7^State Key Laboratory of Brain and Cognitive Sciences, The University of Hong Kong, Hong Kong, China

**Keywords:** Alzheimer-like pathology, age-related macular degeneration, cigarette smoking, retina, amyloid peptide

Alzheimer’s disease (AD) is characterized by the progressive and selective loss of neurons and synapses. This debilitating disease was estimated to affect 33.9 million patients worldwide in 2011, a number that is expected to triple over the next 40 years (1). It has been shown that a combination of several processes, including extracellular deposition of amyloid-beta (Aβ) plaques and the formation of intracellular neurofibrillary tangles (NFTs) composed of hyper-phosphorylated tau proteins, in the brain are involved in the declining cognitive processes associated with AD (2). While dysfunction of the aforementioned biological processes starts from Braak stage I (preclinical AD), it is not until the majority of the neocortex is severely affected by neurofibrillary changes (Braak stages V–VI) that patients are ultimately diagnosed with dementia (3).

Notably, clinical studies have found numerous links between AD and age-related macular degeneration (AMD). AMD is a progressive macular neurodegenerative disease and is the most common cause of irreversible blindness, being estimated to affect 196 million people globally by the year 2020 (4). Although AMD is largely considered a retinal disease, the emerging epidemiological links between cognitive impairment, such as those observed in AD patients, and AMD patients are significant (5). In fact, it appears that cognitive impairment and early AMD may share numerous common age-related pathways and risk factors (6–8). Using logistic regression models that control for age, sex, race, education, systolic blood pressure, total cholesterol level, diabetes mellitus, smoking status, and ApoE genotype, it has been demonstrated that persons with mild cognitive impairment were more likely to have AMD compared to healthy patients (odds ratio of 2.00). It also appears that the reverse is true, with the prevalence of mild cognitive impairment being higher in AMD patients compared to control groups (52.4 vs. 26.8%, *p* < 0.001), with an odds ratio of 3.127 after adjusting for age, education, and visual acuity (8).

The similarities between AD and AMD are not that surprising as the retina is an integral part of the central nervous system (CNS), being derived from the neural tube much like other regions of the brain. AMD and AD also have many parallel characteristics, which have been extensively reviewed by Ohno-Matsui (9). One of the common pathological hallmarks of these two diseases is the extracellular deposits that are highly enriched with Aβ42. In AMD, the deposits, called drusen, are found between the retinal pigmented epithelium (RPE) and Bruch’s membrane. In AD, the deposits found in the brain are referred to as senile plaques and are localized primarily to the hippocampus and cortex. Detailed proteomic analyses of their molecular components have shown similarities between drusen and senile plaques, and proteins such as Aβ, tau, proteoglycans, and inflammatory mediators have been detected in both. These findings suggest that similar pathways could potentially be involved in the etiology of both AMD and AD.

Aβ42 peptides and related amyloidogenic molecules are thought to impact AMD and AD pathology in the following ways: (i) through progressive distortion of the structure and function of the retinal/neocortical architecture; (ii) by promoting mitochondrial dysfunction and the generation of reactive oxygen species (ROS) leading to the oxidation of retinal/neuronal components and apoptosis; (iii) by activating microglial cells, the chief innate-immune “phagocytosis” and “scavenging” cells of the retina/brain and CNS; (iv) by activating microglial-mediated pathological pathways, which have subsequent pro-inflammatory responses. It is important to note that all four of these pathological sequels may occur concurrently during the onset and propagation of AMD/AD (10), and recent research has indicated that AD-like pathology may be accelerated by cigarette smoking (11).

Cigarette smoking (CS) has long been known to be an environmental age accelerator (12). It is estimated that by the year 2020 the number of annual tobacco-related deaths will reach 7.5 million, which will account for approximately 10% of all fatalities (13). According to the 2011 WHO statistics (13), tobacco use was the second most significant risk factor for developing a number of non-transmissible diseases. Interestingly, contrasting reports have been published concerning the associations between CS and the risk of developing AD-like pathology or even AD in different populations. One study indicated that while CS renders protection from AD in Western populations, it appears to increase the risk in Asian populations. After adjusting for heterogeneity, heavy smoking (greater than 55.5 packs per year) does appear to consistently increase the risk of developing AD (14). Various animal studies also suggest that CS induces neuropathological changes that can accelerate the progression of AD (15, 16). Furthermore, in the brain matter of cigarette smoke-exposed Lewis rats, the expression of genes encoding for pro-oxidant iNOS, NOX4, dual oxidase1, and p22phox was increased (17). In the hippocampus of cigarette smoke-exposed SD rats, the oxidative DNA damage marker 8-hydroxyguanosine was also increased (15). Oxidative stress can also activate JNK signaling leading to tau phosphorylation and apoptosis. In fact, after 8 weeks of exposure to cigarette smoke, the level of phospho-JNK was increased along with the levels of tau hyperphosphorylation at residues Thr231, Thr205, and Ser404. Reduced expression of various synaptic proteins (e.g., synapsin-1, synaptophysin) and cytoskeleton-related protein (e.g., tubulin, drebrin) were also detected in these cigarette smoke-exposed rats, indicating possible synaptic degeneration and axonal deficits (15). Furthermore, in a mouse model of AD, cigarette smoke was also observed to exacerbate amyloid pathology, resulting in increased amyloid deposition (which subsequently led to the formation of new plaques) and accelerated maturation of the amyloid deposits after only 4 months of exposure (16).

Compared to the conflicting epidemiological evidence linking AD and CS, CS has been shown to be one of the major modifiable risk factors for AMD, almost doubling the risk of developing AMD while also promoting the progression of the disease from the atrophic to the neovascular form (11, 18, 19). These accelerated changes are likely propagated by the toxic compounds found in cigarette smoke, many of which have been shown to induce oxidative stress and/or decrease free radical scavengers (20). In fact, cigarette smoke extract (CSE) has been shown to significantly reduce the viability of RPE-19 cells and primary RPE cells *via* alterations to mitochondrial integrity and increased lipid peroxidation (21). Moreover, CS promotes molecular and pathological changes that may establish an ideal microenvironment for the development of AMD, vascular inflammation, endothelial dysregulation, oxidative damage, toxic damage, as well as histopathological changes in the RPE, Bruch’s membrane, and choriocapillaris (11). This hypothesis is supported by previous studies showing that 6 months of exposure to cigarette smoke induces changes in these ocular tissues in a mouse model, which mimic the changes that occur in human AMD (22, 23). Importantly, the smoking-related changes leading to accelerated AMD can, to some extent, be reversed when the patient stops smoking if detected early (19).

Clinically, AMD progression is typically monitored by analyzing the deposition of drusen and changes in the pigmentation of the RPE (24). Aβ assemblies are the most prevalent in retinas with moderate-to-high drusen loads in the advanced stages of AMD (25), which potentially already involve irreversible changes in the patient’s vision. While most AMD studies focus on drusen formation in the outer retina, some consideration has been given to early changes occurring in the inner retina. Anti-astrocyte and anti-retinal autoantibodies have been detected in the sera from patients with early forms of AMD, which suggest that changes in the neural retina may also be an early feature of the disease. Abnormalities in the electrooculograms (EOGs) and electroretinograms (ERGs) measured for these retinas also indicate global retinal dysfunction (26). Notably, both normal aging and AMD appear to affect the rod-mediated mfERG measurements (27) and alter neuronal transmission at the postreceptoral level. These functional ERG changes may indicate anatomic and functional plasticity in the synaptic circuity, possibly at the level of photoreceptor-bipolar synapses. In light-damaged rat eyes, which mimic dry AMD, there also appears to be extensive dendritic remodeling and extension of the neurons (28). Moreover, neurons in AMD retinas have the capacity to remodel by sprouting processes and re-forming synaptic complexes with their appropriate targets (29). This remodeling was evident before any evidence of neuronal loss and was accompanied by the reconnection of the presynaptic elements to the postsynaptic bipolar neurons. Thus, if diagnosed early, say before the drusen deposition in the outer retina, AMD could in fact be prevented and/or reversed.

It is in our opinion that in order to diagnose AMD in cigarette smokers early enough to prevent lasting damage, clinicians should focus on the early changes occurring in the retina. To do so, the links between AMD and AD can be exploited to some extent, particularly for the development of diagnostic tools. For example, the techniques presently being developed and optimized to detect structural and functional changes in AD patients could be invaluable for early AMD diagnosis. In fact, the retinas of AD patient have also been shown to be affected by the disease, with Aβ deposition being detected along with retinal ganglion cell degeneration and decreased thickness of the retinal nerve fiber layer (30), suggesting that the inner retina is primary location of damage in AD patients. Although the location of Aβ42 deposition and the cell type affected in the retinas of AMD patients appear to be different compared to AD patients, similar detection tools could be utilized to diagnose AMD before permanent vision loss. It is essential to conduct longitudinal animal studies investigating the neuronal changes of the retina as well as the RPE, Bruch’s membrane, and choidocapillaries together with retinal function tests in order to demonstrate the role these early neuronal changes might also play in AMD pathogenesis. Current animal models can allow us to investigate correlation of these functional changes with the histopathology hallmarks in eyes exposed to CS. Then, we can apply our research findings for non-invasive imaging method to monitor disease progression, contributing to clinical investigations. The information gleaned from these studies may also elucidate additional links between AD and AMD in addition to potentially highlighting biomarkers involved in the early changes occurring during both diseases, in both the brain and retina. Ultimately, we believe it is likely that the link between the brain and retina will play an essential role in aiding researchers in the development of diagnostic tools for CS-induced AMD. The current literature and continued research concerning the early neuronal changes in both the brain and retinas of AD patients and animal models indicate that they could be used as a good model of the early retinal changes in CS-induced AMD. It is our hope that remarking on this phenomenon encourages additional research into the utilization of these previous reports to mediate early diagnosis of AMD (i.e., Aβ, tau, inflammation, microglial activation), and that emerging treatments for AD (anti-amyloid, anti-tau) in conjunction with reduced CS might be useful for the treatment of AMD.

## Author Contributions

SY, RC, and KC wrote the manuscript. RC and KC contribute to the original thought. XT and Y-SH give opinion and discuss the content in the revision processes.

## Conflict of Interest Statement

The authors declare that the research was conducted in the absence of any commercial or financial relationships that could be construed as a potential conflict of interest.

## References

[1] Barnes DE Yaffe K. The projected effect of risk factor reduction on Alzheimer’s disease prevalence. Lancet Neurol (2011) 10(9):819–28. 10.1016/S1474-4422(11)70072-2 21775213 PMC3647614

[2] Wirz KT Keitel S Swaab DF Verhaagen J Bossers K. Early molecular changes in Alzheimer disease: can we catch the disease in its presymptomatic phase? J Alzheimers Dis (2014) 38(4):719–40. 10.3233/JAD-130920 24072070

[3] Braak H Alafuzoff I Arzberger T Kretzschmar H Del Tredici K. Staging of Alzheimer disease-associated neurofibrillary pathology using paraffin sections and immunocytochemistry. Acta Neuropathol (2006) 112(4):389–404. 10.1007/s00401-006-0127-z 16906426 PMC3906709

[4] Wong WL Su X Li X Cheung CM Klein R Cheng CY Global prevalence of age-related macular degeneration and disease burden projection for 2020 and 2040: a systematic review and meta-analysis. Lancet Glob Health (2014) 2(2):e106–16. 10.1016/S2214-109X(13)70145-1 25104651

[5] Zhou LX Sun CL Wei LJ Gu ZM Lv L Dang Y. Lower cognitive function in patients with age-related macular degeneration: a meta-analysis. Clin Interv Aging (2016) 11:215–23. 10.2147/CIA.S102213 26966358 PMC4771401

[6] Baker ML Wang JJ Rogers S Klein R Kuller LH Larsen EK Early age-related macular degeneration, cognitive function, and dementia: the Cardiovascular Health Study. Arch Ophthalmol (2009) 127(5):667–73. 10.1001/archophthalmol.2009.30 19433718 PMC3001290

[7] Johnson LV Leitner WP Rivest AJ Staples MK Radeke MJ Anderson DH. The Alzheimer’s A beta-peptide is deposited at sites of complement activation in pathologic deposits associated with aging and age-related macular degeneration. Proc Natl Acad Sci USA (2002) 99(18):11830–5. 10.1073/pnas.192203399 12189211 PMC129354

[8] Woo SJ Park KH Ahn J Choe JY Jeong H Han JW Cognitive impairment in age-related macular degeneration and geographic atrophy. Ophthalmology (2012) 119(10):2094–101. 10.1016/j.ophtha.2012.04.026 22705343

[9] Ohno-Matsui K. Parallel findings in age-related macular degeneration and Alzheimer’s disease. Prog Retin Eye Res (2011) 30(4):217–38. 10.1016/j.preteyeres.2011.02.004 21440663

[10] Zhao Y Bhattacharjee S Jones BM Hill JM Clement C Sambamurti K Beta-amyloid precursor protein (betaAPP) processing in Alzheimer’s disease (AD) and age-related macular degeneration (AMD). Mol Neurobiol (2015) 52(1):533–44. 10.1007/s12035-014-8886-3 25204496 PMC4362880

[11] Velilla S Garcia-Medina JJ Garcia-Layana A Dolz-Marco R Pons-Vazquez S Pinazo-Duran MD Smoking and age-related macular degeneration: review and update. J Ophthalmol (2013) 2013:895147. 10.1155/2013/895147 24368940 PMC3866712

[12] Nicita-Mauro V Lo Balbo C Mento A Nicita-Mauro C Maltese G Basile G. Smoking, aging and the centenarians. Exp Gerontol (2008) 43(2):95–101. 10.1016/j.exger.2007.06.011 17686596

[13] Global Status Report on Noncommunicable Diseases 2010 – Description of the Global Burden of NCDs, Their Risk Factors and Determinants (2011). World Health Organization Available from: http://www.who.int/nmh/publications/ncd_report2010/en/

[14] Xu W Tan L Wang HF Jiang T Tan MS Tan L Meta-analysis of modifiable risk factors for Alzheimer’s disease. J Neurol Neurosurg Psychiatry (2015) 86(12):1299–306. 10.1136/jnnp-2015-310548 26294005

[15] Ho YS Yang X Yeung SC Chiu K Lau CF Tsang AW Cigarette smoking accelerated brain aging and induced pre-Alzheimer-like neuropathology in rats. PLoS One (2012) 7(5):e36752. 10.1371/journal.pone.0036752 22606286 PMC3350465

[16] Moreno-Gonzalez I Estrada LD Sanchez-Mejias E Soto C. Smoking exacerbates amyloid pathology in a mouse model of Alzheimer’s disease. Nat Commun (2013) 4:1495. 10.1038/ncomms2494 23422663

[17] Khanna A Guo M Mehra M Royal W III. Inflammation and oxidative stress induced by cigarette smoke in Lewis rat brains. J Neuroimmunol (2013) 254(1–2):69–75. 10.1016/j.jneuroim.2012.09.006 23031832 PMC3534934

[18] Chang RC Ho YS Wong S Gentleman SM Ng HK. Neuropathology of cigarette smoking. Acta Neuropathol (2014) 127(1):53–69. 10.1007/s00401-013-1210-x 24240736

[19] Thornton J Edwards R Mitchell P Harrison RA Buchan I Kelly SP. Smoking and age-related macular degeneration: a review of association. Eye (Lond) (2005) 19(9):935–44. 10.1038/sj.eye.6701978 16151432

[20] Church DF Pryor WA. Free-radical chemistry of cigarette smoke and its toxicological implications. Environ Health Perspect (1985) 64:111–26. 10.1289/ehp.8564111 3007083 PMC1568603

[21] Bertram KM Baglole CJ Phipps RP Libby RT. Molecular regulation of cigarette smoke induced-oxidative stress in human retinal pigment epithelial cells: implications for age-related macular degeneration. Am J Physiol Cell Physiol (2009) 297(5):C1200–10. 10.1152/ajpcell.00126.2009 19759330 PMC2777395

[22] Fujihara M Nagai N Sussan TE Biswal S Handa JT. Chronic cigarette smoke causes oxidative damage and apoptosis to retinal pigmented epithelial cells in mice. PLoS One (2008) 3(9):e3119. 10.1371/journal.pone.0003119 18769672 PMC2518621

[23] Espinosa-Heidmann DG Suner IJ Catanuto P Hernandez EP Marin-Castano ME Cousins SW. Cigarette smoke-related oxidants and the development of sub-RPE deposits in an experimental animal model of dry AMD. Invest Ophthalmol Vis Sci (2006) 47(2):729–37. 10.1167/iovs.05-0719 16431974

[24] Bird AC Bressler NM Bressler SB Chisholm IH Coscas G Davis MD An international classification and grading system for age-related maculopathy and age-related macular degeneration. The International ARM Epidemiological Study Group. Surv Ophthalmol (1995) 39(5):367–74. 10.1016/S0039-6257(05)80092-X 7604360

[25] Anderson DH Talaga KC Rivest AJ Barron E Hageman GS Johnson LV. Characterization of beta amyloid assemblies in drusen: the deposits associated with aging and age-related macular degeneration. Exp Eye Res (2004) 78(2):243–56. 10.1016/j.exer.2003.10.011 14729357

[26] Walter P Widder RA Luke C Konigsfeld P Brunner R. Electrophysiological abnormalities in age-related macular degeneration. Graefes Arch Clin Exp Ophthalmol (1999) 237(12):962–8. 10.1007/s004170050331 10654164

[27] Feigl B Brown B Lovie-Kitchin J Swann P. The rod-mediated multifocal electroretinogram in aging and in early age-related maculopathy. Curr Eye Res (2006) 31(7–8):635–44. 10.1080/02713680600762739 16877272

[28] Sullivan R Penfold P Pow DV. Neuronal migration and glial remodeling in degenerating retinas of aged rats and in nonneovascular AMD. Invest Ophthalmol Vis Sci (2003) 44(2):856–65. 10.1167/iovs.02-0416 12556422

[29] Sullivan RK Woldemussie E Pow DV. Dendritic and synaptic plasticity of neurons in the human age-related macular degeneration retina. Invest Ophthalmol Vis Sci (2007) 48(6):2782–91. 10.1167/iovs.06-1283 17525213

[30] Heaton GR Davis BM Turner LA Cordeiro MF Ocular biomarkers of Alzheimer’s disease. Cent Nerv Syst Agents Med Chem (2015) 15(2):117–25. 10.2174/1871524915666150319123015 25788142

